# Tracking of Tobacco Mosaic Virus in Taxonomically Different Plant Fungi

**DOI:** 10.3390/jof11090619

**Published:** 2025-08-25

**Authors:** Natascia Filomena Barnaba, Lorenza Vaccaro, Rita Milvia De Miccolis Angelini, Roberta Spanò, Franco Nigro, Tiziana Mascia

**Affiliations:** 1 Department of Soil, Plant and Food Sciences, University of Bari Aldo Moro, 70126 Bari, Italy; natascia.barnaba@uniba.it (N.F.B.); lorenza.vaccaro@uniba.it (L.V.); ritamilvia.demiccolisangelini@uniba.it (R.M.D.M.A.); franco.nigro@uniba.it (F.N.); 2 Institute for Sustainable Plant Protection (IPSP), National Research Council, 70126 Bari, Italy; roberta.spano@cnr.it

**Keywords:** *Botrytis cinerea*, *Fusarium oxysporum*, *Monilinia fructicola*, RNAi, TMV-based recombinant vector, *Verticillium dahliae*

## Abstract

Plant viruses have been traditionally considered pathogens restricted to plant hosts. However, recent studies have shown that some plant viruses can infect and replicate in filamentous fungi and oomycetes, suggesting that their host range is broader than previously thought, and that their ecological interactions are more complex. In this study, we investigated the ability of the well-characterized positive-sense RNA plant virus Tobacco mosaic virus (TMV) to replicate in four major phytopathogenic fungi from different taxonomic groups: *Botrytis cinerea*, *Fusarium oxysporum* f. sp. *lycopersici*, *Verticillium dahliae*, and *Monilinia fructicola*. Using a recombinant TMV-based vector expressing a green fluorescent protein (TMV-GFP-1056) as reporter, we demonstrated that TMV can enter, replicate, and persist within the mycelia of *B. cinerea* and *V. dahliae*—at least through the first subculture. However, it cannot replicate in *F. oxysporum* f. sp. *lycopersici* and *M. fructicola*. RNA interference (RNAi) is a conserved eukaryotic epigenetic mechanism that provides an efficient defence against viruses. We explored the role of RNAi in the interaction between TMV and the mycelia of *V. dahliae* and *B. cinerea*. Our results revealed a strong induction of the *Dicer-like 1* and *Argonaute 1* genes, which are key compounds of the RNA silencing pathway. This RNAi-based response impaired TMV-GFP replication in both fungi. Notably, despite viral replication and RNAi activation, the virulence of *V. dahliae* and *B. cinerea* on their respective host plants remained unaffected. These findings reinforce the emerging recognition of cross-kingdom virus transmission and interactions, which likely play a crucial role in pathogen ecology and viral evolution. Understanding these virus–fungus interactions not only sheds light on RNAi interference silencing mechanisms but also suggests that plant viruses like TMV could serve as simple and effective tools for functional genomic studies in fungi, such as in *V. dahliae* and *B. cinerea*.

## 1. Introduction

Plant viruses are a highly diverse and abundant group of pathogens responsible for numerous diseases that impact global agriculture [1]. Despite extensive research efforts, their genetic diversity and ecological distribution remain only partially elucidated [2]. Traditionally, plant viruses are known to infect and replicate mainly within plant hosts. However, recent evidence suggests that some plant viruses can also replicate in insect vectors, especially aphids and whiteflies, which are critical agents for virus transmission and epidemiological dissemination of many significant crop diseases [3,4,5].

Transmission via soil-borne vectors, such as nematodes and fungi, has been documented, although replication in these environments occurs less frequently. Recent metagenomic and high-throughput sequencing studies have identified viral sequences in non-plant organisms, including bats and various insects [6] as well as fungi and oomycetes [7], suggesting the potential for occasional cross-kingdom viral infections in natural ecosystems, thus challenging the traditional concept of host specificity.

Cross-kingdom infections, although often transient or non-persistent, indicate that fungi may actively participate in the life cycles, transmission, and evolution of viruses [8]. Experimental evidence supports the bidirectional transfer of viruses between plants and fungi, a process enabled by their close ecological relationships. This paradigm challenges the traditional view of strict viral host specificity and reveals a more complex viral ecology, with significant implications for disease management and our understanding of viral evolution [7,9]. The ecological and agricultural significance of fungal pathogens such as *Botrytis cinerea*, *Fusarium oxysporum* f. sp*. lycopersici* (*Fol*), *Verticillium dahliae*, and *Monilinia fructicola* reflects their role in plant health, crop productivity, and ecosystem functionality. These fungal pathogens are major agricultural challenges, causing significant yield losses. Their impacts extend beyond crop health, shaping ecological interactions and influencing food security globally. *B. cinerea* which causes grey mould in fruits and vegetables, thrives in high-moisture environments, producing secondary metabolites that enhance its pathogenicity [10]. This fungus leads to major economic losses, particularly in grapes and soft fruits, though innovative biocontrol measures show promise in mitigating these impacts [11,12,13]. *V. dahliae*, a soil-borne pathogen, causes wilting and death in numerous crops. Its ability to persist for many years in soil via resistant microsclerotia complicates management, requiring integrated pest control strategies [14,15]. The evolution and the dissemination of virulent strains (i.e., defoliating pathotypes) present further challenges, especially in resistance management, with specific microRNAs helping crops like cotton enhance defence [16]. Similarly, Fol causes *Fusarium* wilt in tomato and is notoriously difficult to control due to its soil-borne nature and wide distribution [17]. Its high genetic diversity and production of toxic metabolites pose risks not only to plants but potentially to humans consuming contaminated produce [18]. Effective soil health management is critical for controlling *Fusarium*’s impact. *M. fructicola* is the primary agent of brown rot in stone fruits, significantly impacting peaches, cherries, and plums [19]. It disperses widely through aerial spores and thrives in warm, humid conditions. Resistance mutations from excessive fungicide use highlight the need for sustainable practices, such as developing resistant cultivars and using biological control agents [19].

These fungi belong to different *Ascomycota* lineages: *B. cinerea* and *M. fructicola* (family *Sclerotiniaceae*, class *Leotiomycetes*) are necrotrophs mainly affecting fruit post-harvest [20,21] whereas *V. dahliae* and Fol (class *Sordariomycetes*, families *Plectosphaerellaceae* and *Nectriaceae*) cause systemic wilting, impacting plant yield and longevity [17,18].

Ecologically, these pathogens influence nutrient cycling, plant community dynamics, and agroecosystem function. Soil fungal communities impact plant health by shaping the microbiome, enhancing resilience against pathogens [22,23]. In this context, understanding the interactions between plant viruses and plant-associated fungi could provide novel insights into pathogen ecology or potential disease management strategies [12,24].

Recent findings reveal that plant-associated fungi can naturally acquire plant viruses in a non-persistent manner, suggesting a possible involvement in the life cycles and evolution of viruses [13]. Notably, six taxonomically different plant viruses have been shown to replicate and persist within *Phytophthora infestans* (an oomycete pathogen infecting potato) [25,26] and in *Colletotrichum acutatum* [27]. Brants et al. [28] demonstrated that Tobacco mosaic virus (TMV) can enter and persist within the mycelia of soil-borne oomycetes such as *Pythium* species. Additionally, tobacco necrosis virus particles have been experimentally introduced into *Pythium aphanidermatum* [29]. TMV, the first virus ever discovered, primarily infects plants in the *Solanaceae* family, including tobacco (*Nicotiana tabacum*), tomato (*Solanum lycopersicum*), and pepper (*Capsicum* spp.) [30,31,32,33]. TMV is a single-stranded, positive-sense RNA virus with a genome approximately 6.4 kb in length, encoding proteins essential for replication, movement, and encapsidation [34]. Due to its high stability and extensive study, TMV is widely used in genetic manipulation, making it a valuable tool in biotechnology and functional genomics research. [35,36]. In this study, we aimed to investigate whether *B. cinerea*, *V. dahliae*, *M. fructicola* and *Fol*, belonging to different taxa and with diverse physical and structural characteristics (such as life cycle, infection site, and cell wall composition), could serve as suitable hosts for TMV. Taxonomic and functional diversity could allow for comparative studies of viral interactions with fungi using different infection strategies, such as necrotrophy and vascular wilting. To explore these cross-kingdom interactions, we used a TMV-based vector expressing a green fluorescent protein (TMV-GFP-1056). More importantly, we aimed to assess its suitability for functional genomics applications by employing virus-induced gene silencing (VIGS) strategies. Previous studies have shown that TMV-based vectors can infect *Colletotrichum* species, enabling both reporter gene expression and gene silencing, underscoring their potential as a fungal gene expression system [27].

Building on this, we used a modified TMV-based vector that retains its native replication and movement genes but also includes an additional subgenomic RNA promoter to drive the expression of a target protein-coding gene. This system enables the high-level expression of free, soluble proteins in infected cells. The TMV vector employs a duplicated subgenomic promoter system to ensure the coordinated and robust transcription of foreign genes. Unlike viral vectors, which integrate foreign DNA into the host genome, this TMV-based system operates exclusively in the cytoplasm, producing large amounts of messenger RNA (mRNA) without altering the host’s genetic material [37].

As a result, this vector does not constitute genetic modification of the host plant and is considered substantially safe. To further mitigate potential risks, the vector includes modifications designed to limit homologous recombination. This incorporates a duplicated subgenomic RNA promoter derived from Tobacco mild green mosaic virus (TMGMV), a related tobamovirus associated with milder symptoms than TMV. This modification helps mitigate disease severity during infection [37,38].

A better knowledge of the mechanisms of TMV infection may reveal new vulnerabilities within fungal pathogens, offering potential targets for the development of new control approaches [27,39]. By utilizing recombinant TMV vectors, it could be possible to target fungal species by expressing antifungal proteins or triggering immune responses within the fungal pathogens. These viral vectors could be customized to deliver functional genes that inhibit fungal growth or reduce the virulence of these pathogens [40,41]. This knowledge may reveal new vulnerabilities within fungal pathogens, offering potential targets for the development of biocontrol agents. For example, exploiting TMV-based vectors as a method of introducing antifungal genes into the fungi could provide a new avenue for controlling fungal infections in crops [26,27].

Post-transcriptional gene silencing (PTGS), also known as RNA interference (RNAi), is a conserved eukaryotic mechanism that regulates gene expression by degradation sequence-specific RNA and serves as an antiviral defence system [42]. In fungi, such as *B. cinerea* and *V. dahliae* [41,43], RNAi and the related quelling process involve RNA-dependent RNA polymerases (RdRp), *Dicer-like* (DCL) enzymes, and *Argonaute* (AGO) proteins. These components generate small interfering RNAs (siRNAs) that guide the degradation of complementary messenger RNAs (mRNAs) [44,45]. The RNAi machinery has been extensively studied in *B. cinerea* and *V. dahliae* [41,43], where it plays a crucial role in modulating pathogenicity, virulence, and stress responses. Furthermore, RNAi has emerged as a promising strategy for disease control in *B. cinerea* and *V. dahliae* [39,40,41]. Notably, *B. cinerea* can incorporate small RNAs (sRNA) derived from host plants through inter-kingdom RNAi, which influences its virulence [41]. Similarly, *V. dahliae* engages in inter-kingdom RNAi interactions, offering potential new avenues for innovative crop protection methods [46]. Key RNAi components have also been identified in Fol, indicating the presence of functional RNAi pathways that are likely to be involved in gene regulation and antiviral defence [47,48]. Research on RNAi mechanisms in *M. fructicola* is ongoing [49]. Here, we report the first evidence that a recombinant vector derived from a positive-sense RNA plant virus can enter, replicate, and persist in the plant-associated fungi *B. cinerea* and *V. dahliae*, despite their differences in taxonomy, morphology, and host interactions. Furthermore, these fungi activate RNAi responses upon TMV infection. Interestingly, TMV-GFP failed to infect *F. oxysporum* f. sp. *lycopersici* and *M. fructicola*, suggesting the existence of host-specific barriers to viral infection.

The research into TMV infections in these four fungi holds the potential to transform fungal control strategies [39]. By uncovering novel viral mechanisms, enhancing biocontrol strategies, and targeting fungal resistance mechanisms, it could be possible to develop new, effective, and sustainable ways to manage these agricultural pathogens, reducing reliance on harmful chemicals and fostering more resilient agricultural practices [20].

## 2. Materials and Methods

### 2.1. TMV-GFP-1056 Recombinant Vector and Plant Materials

TMV-green fluorescent protein (GFP) inoculum was maintained in young *Nicotiana benthamiana* plants, which were rub-inoculated with biologically active transcripts synthesized using a T7 RNA polymerase mMessage mMachine kit (Ambion) from 2 μg of *KpnI*-linearized pBSG1056, following the manufacturer’s protocol. The plasmid pBSG1057 contains the GFP open reading frame (ORF) placed under the control of the TMV coat protein (CP) of Tobacco mild green mosaic virus (TMGMV) subgenomic mRNA promoter [27,50]. Tobacco virus-free mock-inoculated plants served as negative controls (six plants were used per experiment). All plants were grown in chambers at 22 ± 2 °C, with 70–80% humidity and a 16 h/8 h (day/night) photoperiod. Plants were monitored weekly for the GFP expression using a B100-AP Black Ray long-wave UV lamp (UVP). Systemically infected leaves, at 14 dpi, exhibiting GFP fluorescence and showing clear viral symptoms, were crushed in 100 mM Na_2_/K phosphate buffer (pH 7.2), and the plant sap was quickly filtered using 0.22 μm cellulose filters. The filtered sap (1 mL) was added to 10^6^ conidia/mL cultures of *B. cinerea*, Fol, *V. dahliae*, and *M. fructicola* grown in 150 mL of liquid medium containing 50 mg/mL streptomycin sulphate to prevent bacterial contamination, following the procedures described by Mascia et al. [27]. The cultures were maintained under continuous shaking at 150 rpm. Liquid cultures of *B. cinerea*, Fol, *V. dahliae*, and *M. fructicola* inoculated with healthy *N. benthamiana* sap served as negative controls. The assay was performed twice.

### 2.2. Fungal Isolates and Culture Conditions

The following fungal species were used in this study: *B.cinerea* strain SAS56 (Family: Sclerotiniaceae; Genus: *Botryotinia*) [51], *M. fructicola*, (CBS 144850) strain Mfrc123 (Family: Sclerotiniaceae; Genus: *Monilinia*) [16], Fol CBS123668 (Family: Nectriaceae; Genus: *Fusarium*) [18] and the non-defoliating isolate V10 of *V. dahliae* (Family: Incertaesedis; Genus: *Verticillium*), which belongs to the *V. dahliae* collection of the Department of Soil, Plant and Food Sciences (DISSPA), University of Bari “Aldo Moro” [52].

All strains were stored in 10% glycerol at −80 °C and refreshed on potato dextrose agar (PDA: 200 g peeled and sliced potatoes infused for 1 h at 60 °C, 20 g dextrose, pH 6.5, and 20 g LLG-European bacteriological agar per litre of distilled water). The *V. dahliae* isolate was grown on PDA for 15 days, after which agar plugs from the colony were used to inoculate two 250 mL Erlenmeyer flasks, each containing 100 mL of carrot broth (CB: 200 g peeled and sliced carrots infused for 1 h at 60 °C, 20 g dextrose, per litre of distilled water, pH 6.5). Cultures were then incubated at 25 °C in the dark on an orbital shaker at 150 rpm for 2 days. A conidial suspension was prepared by filtering the entire culture broth through two layers of sterile cheesecloth, and the concentration was adjusted to 1 × 10^6^ conidia/mL using a hemocytometer. To induce sporulation, *B. cinerea* colonies were maintained at 21 ± 1 °C in darkness for 2 days, followed by exposure to 12 h/day to a combination of two daylight lamps (Osram L36W/640) and two near-UV lamps (Osram L36/73) for 7 days [53]. *M. fructicola* and Fol strains were grown at 25 ± 1 °C in darkness for 8 days. Conidia from all three fungal strains were harvested by scraping the sporulating colonies in sterile distilled water containing 0.1% Tween 20. The suspensions were then filtered through Miracloth (Calbiochem, San Diego, CA, USA) to remove hyphal fragments, and the conidial concentration was adjusted to 1 × 10^6^ conidia/mL. Minimal medium (MM: 10 mL solution A, 10 mL solution B, 1 mL micronutrient solution, 20 g glucose per litre of distilled water) was used for *B. cinerea* and *M. fructicola*, whereas Fol and *V. dahliae* were grown in Czapek-Dox broth (C1551, Millipore, Darmstadt, Germany), supplemented with 50 mg/mL streptomycin sulphate.

### 2.3. RNA Extraction and Subculturing Conditions

Mycelia were harvested from liquid cultures at 6, 9, and 13 days post-inoculation (dpi) and subjected to a 45 s treatment with a 2% (*v*/*v*) commercial sodium hypochlorite solution [34,35,36] to effectively degrade virus particles adhering to the external surfaces of the hyphae, then washed extensively with sterile distilled water. For RNA extraction, 100 mg of freeze-dried mycelia ground to a fine powder with liquid nitrogen were processed with TRIzol reagent (cat. no. T3934, Sigma-Aldrich, St. Louis, MO, USA), according to the manufacturer’s instructions. Sampling at 6, 9, and 13 dpi was based on established TMV infection dynamics in *N. benthamiana* plants (its natural hosts), where early replication occurs within 6 dpi and systemic accumulation typically peaks around 10–14 dpi. These intervals allowed for monitoring potential viral replication and RNAi responses in fungal mycelia across early, intermediate, and late stages post-inoculation [43,54]. For subculturing, the first subculture (S1) was produced by transferring mycelia from liquid cultures onto PDA plates and incubating at 25 ± 1 °C in the dark for 10 days, then collected for RNA extraction. Mycelial growth of the first subcultures, expressed in mm/day, was recorded daily for 10 days. Data from five replicates were used to calculate the average macroscopic and microscopic morphological characteristics. The assay was repeated twice, including a mock-inoculated control.

### 2.4. Optical and Confocal Microscopy

Microscopy observations were performed on *B. cinerea*, *V. dahliae*, Fol and *M. fructicola* mycelia, collected at 9 dpi. Fluorescence images were acquired using a ViCo-equipped Nikon Eclipse 80i confocal microscope (Nikon Instruments Inc., Melville, NY, USA). Bright-field images were captured from the same regions.

### 2.5. Virus Detection and Analysis

Total RNA was treated with 1 U/µg RNA of RQ1 RNase-free DNase (Promega Corporation, Madison, WI, USA) for 30 min at 37 °C, following the manufacturer’s protocol, and subsequently dissolved in nuclease-free water. RNA concentration and purity were assessed using Nanodrop ND-1000 (Thermo FisherScientific, Waltham, MA, USA) and Qubit 2.0 fluorometer (Life Technologies Ltd., Paisley, UK). For cDNA synthesis, 500 ng of total RNA was reverse transcribed with High-Capacity cDNA Reverse Transcription Kit (Thermo Fisher Scientific Inc., Wilmington, DE, USA). Standard reverse-transcription polymerase chain reactions (RT-PCR) were employed to detect the green fluorescent protein (GFP), TMV replicase and coat protein (CP) genes in mycelia of *B. cinerea*, *V. dahliae*, Fol and *M. fructicola.* The purity and size of the amplified products were assessed by electrophoresis on a 1.2% agarose gel in TBE (90 mM Tris, 90 mM boric acid, 1 mM EDTA, pH 8) buffer, followed by Gel-red staining.

To evaluate virus accumulation, RT-qPCR assays were performed on three biological replicates of *V. dahliae* and *B. cinerea* samples collected at 6-, 9-, and 13-dpi using the StepOne apparatus (Applied Biosystems, Thermo Fisher Scientific) and the PowerUp™ SYBR™ Green Master Mix (Thermo Fisher Scientific). The transcription expression level of genes encoding components of the silencing pathway was quantified in the same samples using cDNA from virus-infected and mock-inoculated fungal isolates collected at the same time points. No-template controls were used to detect nonspecific amplification. The assay was repeated twice.

Relative quantification (RQ) of *Dicer-like 1* (DCL1) (Acc. n. XM_009651848.1) and *Argonaute 1* (AGO1) (Acc. n. XM_009659642.1) in *V. dahliae* and of DCL1 (Acc. n. A0A384JZT2) and AGO1 (Acc. n. A0A384JB02) in *B. cinerea* was estimated using 2 µL of cDNA (corresponding to 50 ng of total RNA) and 1.2 µL of each primer (2.5 µM). The *actin* (Acc. N. XM_001553318.1) [55] and *β-tubulin* genes (Acc. N. G2XIV1) [46,56] were used as internal reference genes for *B. cinerea* and *V. dahliae*, respectively. Relative gene expression was calculated using the comparative Ct method (2^−ΔΔCt^) [57]. Each experiment included three biological replicates. The assay was repeated twice.

For virus detection in subculture S1 of *V. dahliae* and *B. cinerea*, RNA samples underwent dot blot hybridization with digoxigenin (DIG)-labelled DNA probes specific for TMGMV CP following the protocol by Minutillo et al. [58]. The same first subcultures that were positively transfected with TMV-GFP were used to perform pathogenicity tests.

When necessary, RNA preparations were separated by electrophoresis on 1.2% agarose gels in TBE buffer, stained with GelRed^®^ (Biotium, Fremont, CA, USA), and transferred onto positively charged nylon membranes. Northern blot hybridizations were performed using DIG-labelled DNA probes specific for TMGMV CP (for *B. cinerea*) and TMV RdRp (for *V. dahliae*). DIG-labelled DNA probes were synthesized using the PCR DIG Probe Synthesis Kit (Roche Diagnostics Corporation, Indianapolis, IN, USA).

To detect vsiRNAs, RNA was separated on 15% denaturing polyacrylamide gels and electroblotted onto nylon membranes at 15 V for 1 h in 0.5× TBE. UTP-DIG-labelled CP probe was transcribed from corresponding sequences in the pBSG1057-TMV plasmid. RNA probes were hydrolyzed by adding 300 µL of 200 mM carbonate buffer (80 mM NaHCO_3_, 120 mM Na_2_CO_3_) to 20 µL of probe solution and incubating at 60 °C for 3 h. The hydrolysis reaction was stopped by adding 20 µL of 3 M sodium acetate (pH 5.0). Hybridization and detection were performed following the procedures described by Skepper et al. [59]. Synthetic 21-nt DNA oligonucleotides served as size markers. Gel electrophoresis, blotting, hybridization, and detection were conducted according to the DIG Application Manual (Roche). Chemiluminescent signals were detected using the ChemiDoc imaging system and Image Lab software version 5.1. Bio-Rad Laboratories, Hercules, CA, USA).

Primer pairs are listed in the Supplementary Information (SI), Table S1.

### 2.6. Pathogenicity Assays

We evaluated the effect of TMV-GFP infection on the pathogenicity of *B. cinerea* in strawberry and cucumber, and of *V. dahliae* in tomato, respectively.

For the *B. cinerea* pathogenicity assay, organic strawberry fruits (cv. Candonga’) and cucumber cotyledons (cv. Mezzo Lungo di Polignano’) were artificially inoculated with TMV-GFP and with mock control mycelium. Before inoculation, fruits and cotyledons were surface-decontaminated by immersion in 2% sodium hypochlorite for 1 min, rinsed twice with sterile distilled water, and air-dried at room temperature. Strawberry fruits were punctured with a sterile needle (5 mm diameter in depth) after being inoculated with 15 µL of 1 × 10^5^ conidia mL^−1^ conidial suspensions preliminarily prepared in sterile distilled water containing 0.01% Tween 20 from 8-day-old PDA cultures and filtered through Miracloth (Calbiochem, San Diego, CA, USA). Cucumber cotyledons were placed on Water Agar (WA; 6 g of agar per litre of distilled water) plates (90 mm diameter), wounded (1 mm diameter in depth), and inoculated with a 4 mm diameter mycelium PDA plug. Fruits inoculated with sterile distilled water containing 0.01% Tween 20 and cotyledons inoculated with sterile PDA plugs served as mock controls. After inoculation, samples were incubated in a moist chamber at 21 ± 1 °C in the dark. Each treatment consisted of three biological replicates and each biological replicate was used to inoculate 10 fruits or 5 cotyledons (technical replicates). The assay was repeated twice.

To test virus infectivity in *S. lycopersicum* cv. Marmande plants, either mock-inoculated or TMV-GFP-infected *V. dahliae* mycelia, were grown on PDA at 24 ± 1 °C for 15 days. Conidia were then collected by scraping sporulating colonies into phosphate-buffered saline buffer (PBS; 137 mM NaCl, 2.7 mM KCl, 10 mM Na_2_HPO_4_, 1.8 mM KH_2_PO_4_, pH 7.4), filtered through Miracloth to remove any mycelium fragments, and adjusted to a concentration of 1 × 10^7^ conidia/mL using a hemocytometer. One-month-old potted tomato plantlets were water-stressed for one week before inoculation. Plants (N = 7 per treatment) were inoculated by injecting 15 µL of the conidial suspension into the base of the stem using an insulin syringe (0.3 mL; 31G × 8 mm, Pic Solution, Medical Technology and Devices S.p.A., Casnate con Bernate, Italy). Control plants (N = 7) were inoculated with 15 µL of PBS buffer. Plantlets were then maintained in a greenhouse at 27 ± 2 °C for 2 months. Disease progression was assessed using the McKinney index (Σ (C × F) × 100/(N × v)), where C = disease class, F = number of plants in class, N = total plants, and v = highest class value [17,60,61].

Experiments were conducted using three biological replicates (independent mycelial cultures) per treatment; each biological replicate was used to inoculate seven individual plants (technical replicates). All experiments were independently repeated two times.

### 2.7. Statistical Analyses

Variables were tested for normality using the Shapiro–Wilk test, and for homogeneity of variance using the Levene test (Squared Deviations). One-way ANOVA followed by Tukey’s Honestly Significant Difference (HSD) post hoc test was used to assess differences between groups. In the case of the pathogenicity test of *V. dahliae* on tomatoes where the assumptions of normality and homogeneity of variances were not met, the non-parametric Mann–Whitney U test was applied to compare differences between groups. Statistical significance was considered at *p* < 0.05. Data analysis was performed by OriginPro (version 2023) (OriginLab corporation, Northampton, MA, USA).

## 3. Results

### 3.1. TMV Enters and Replicates in Mycelia of Botrytis and Verticillium spp., but Not in Fusarium and Monilinia spp.

**Figure 1:**
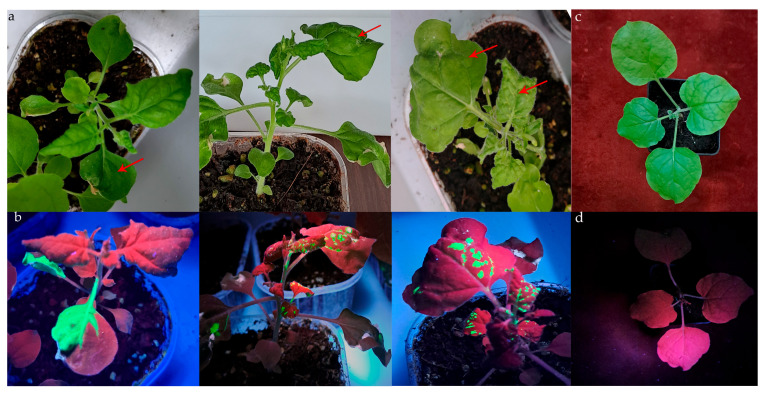
Assessment of TMV-GFP-infected young plants at 14 dpi in *N. benthamiana* plants (**a**) Symptoms of TMV-infected plants include leaf curling and epinasty under visible light. Red arrows in panel (**a**) indicate the same leaf area of *N. benthamiana* plants inoculated with TMV-GFP emitting green fluorescence when exposed to UV light. (**b**) Mock-inoculated tobacco plants under visible (**c**) and UV light (**d**), used as negative control.

In a preliminary investigation, we evaluated the ability of TMV-GFP to enter, replicate and express in four taxonomically different species of phytopathogenic fungi: Fol CBS123668, *V. dahliae* V10, *M. fructicola* Mfrc123, and *B. cinerea* SAS56. Sap from *N. benthamiana* systemically TMV-GFP-infected leaves showing green fluorescence, used as a reporter, and with viral symptoms (Figure 1a), was added to liquid cultures containing 10^6^ conidia/mL suspension of each fungal isolate. Thirty mycelial samples were randomly collected at 6, 9, and 13 dpi for molecular assays. Part of the mycelia was transferred individually onto a solid medium for 10 days to obtain the first subculture. The same isolates challenged with sap from mock-inoculated plants were used as controls.

**Figure 2:**
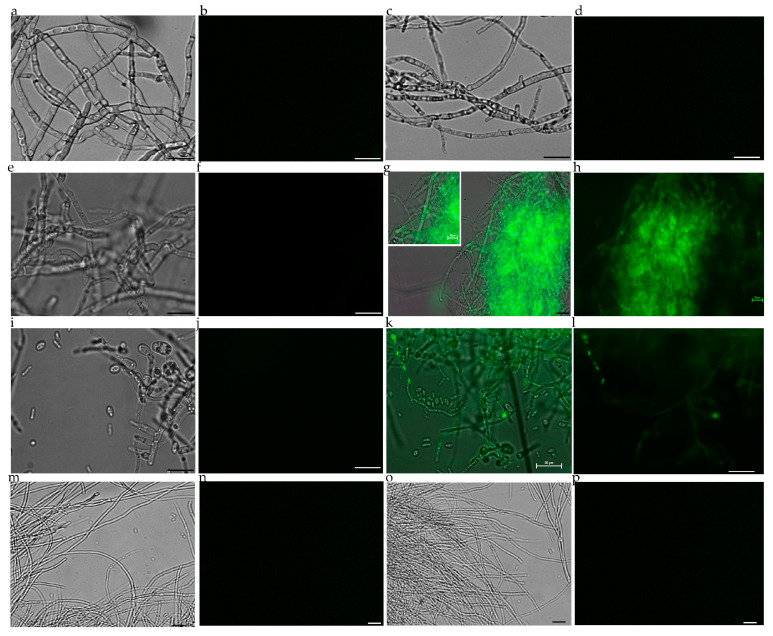
The tobacco mosaic virus-based recombinant vector TMV-GFP-1056 is expressed in *V. dahliae* and *B. cinerea*, but not in Fol and *M. fructicola.* Images of WT *M. fructicola* not exposed to viral co-incubation and served as negative control (**a**,**b**). Absence of epifluorescence in hyphae of *M. fructicola* with TMV-GFP-1056 under white (**c**) and UV light (**d**). Absence of epifluorescence *B. cinerea* mycelia not exposed to co-incubation with TMV-GFP and served as a control (**e**,**f**). Uniform epifluorescence and confocal microscopy of *B. cinerea* with TMV-GFP viewed under UV light (**h**) and merged (**g**). Images of *V. dahliae* uninfected controls viewed under white (**i**) and UV light (**j**). Epifluorescence and confocal microscopy of *V. dahliae* with TMV-GFP with green fluorescence under UV (**l**) and merged (**k**). Images of WT Fol not exposed to viral co-incubation (**m**,**n**). Absence of epifluorescence in hyphae of Fol with TMV-GFP-1056 under white (**o**) and UV (**p**) light. (**a**,**c**,**e**,**i**,**m**,**o**) and (**b**,**d**,**f**,**j**,**n**,**p**) show the same samples viewed under white and UV light, respectively. (**g**,**k**) and (**h**,**l**) show the same sample viewed under merged and UV light, respectively. Scale bars 20 μm.

Confocal microscopy observations of mycelia inoculated with TMV-GFP collected at 13 dpi and treated with sodium hypochlorite to remove externally adhering virus particles, revealed a strong and diffuse green fluorescence in hyphae and conidia of *B. cinerea* (Figure 2h) and *V. dahliae* (Figure 2l), but not in *M. fructicola* (Figure 2d) and Fol (Figure 2p), as well as also in mycelia not exposed to viral inoculum used as negative control (Figure 2a,e,i,m). These findings are consistent with previous reports [25,26,27] and indicate a relatively uniform distribution and expression of the recombinant virus vector in both *V. dahliae* and *B. cinerea*. This is in line with the virus’s inability to enter and express within *Fusarium* and *Monilinia* spp. A preliminary assessment of the virus’s ability to enter and replicate in filamentous fungi and to achieve ectopic protein expression within their cells is necessary for future functional genomics studies.

**Figure 3:**
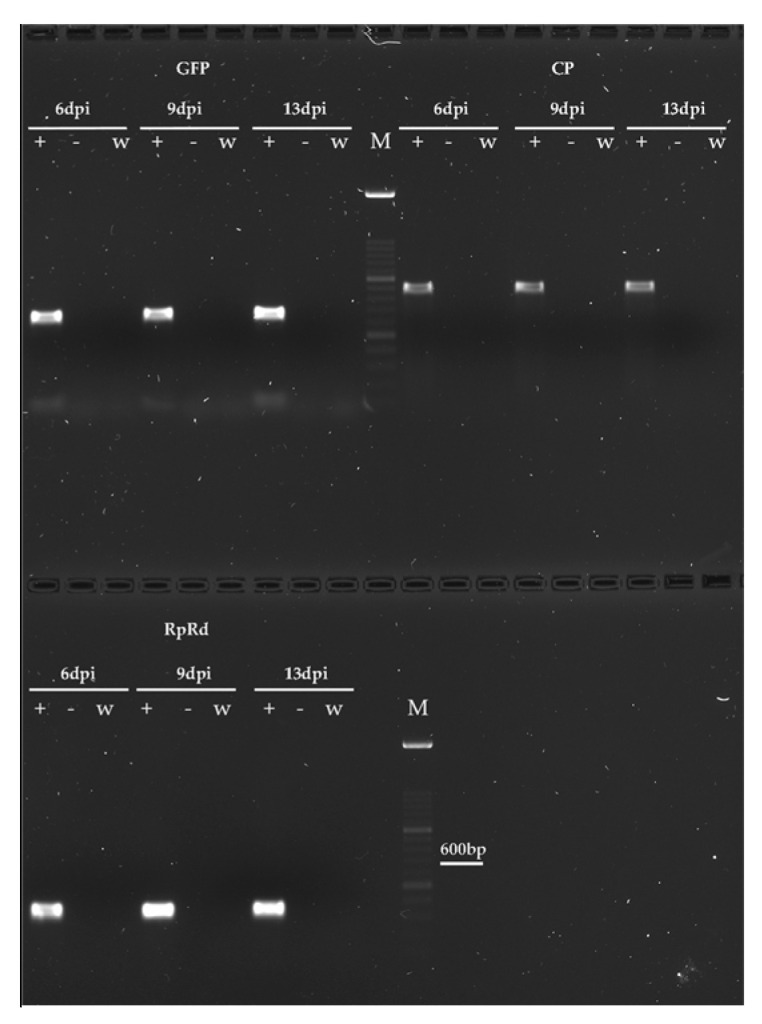
Detection of plant virus infection in mycelia of *B. cinerea* by RT-PCR. Amplicons of the expected size were obtained in preparations of total RNA from mycelium of *B. cinerea* collected at 6, 9 and 13 dpi with TMV-GFP. PCR assay of GFP, CP and RdRp of TMV yielded the expected amplicons of 466 bp, 643 bp and 241 bp, respectively. Amplicons were absent in mock-inoculated mycelia of *B. cinerea*, used as negative control; M = HyperLadder^TM^ 100bp (Bioline, Meridian Bioscience Inc., Cincinnati, OH, USA); w = white control (no cDNA); − = negative control from mock-inoculated mycelia; + = *B. cinerea* inoculated with TMV-GFP.

**Figure 4:**
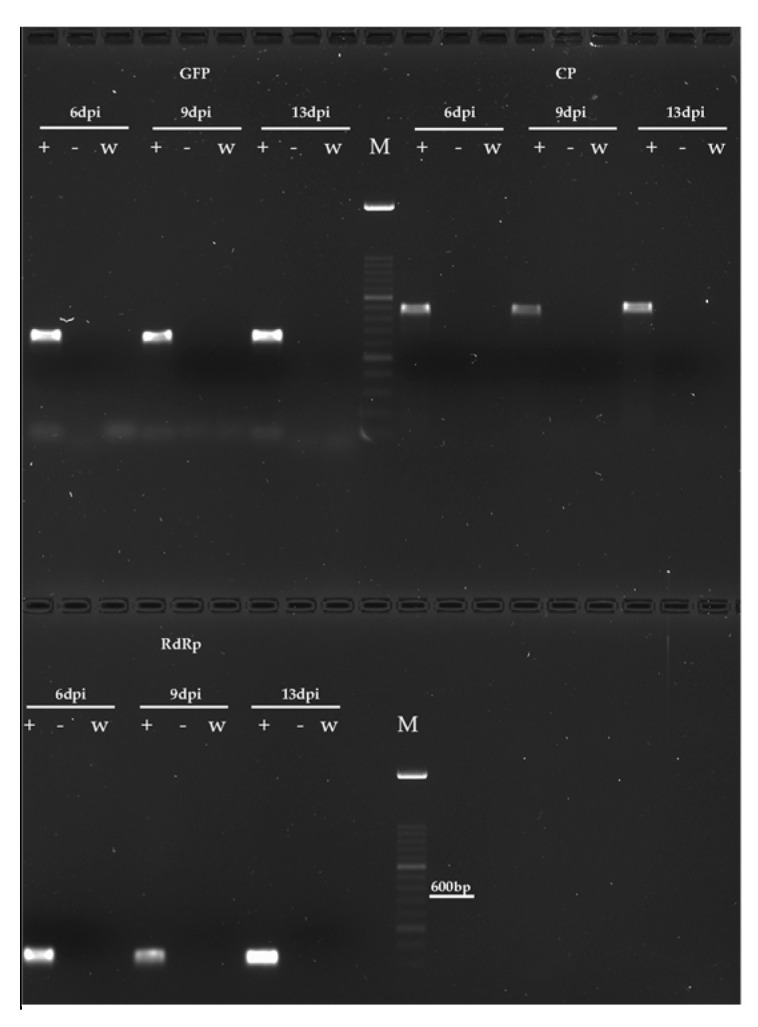
Molecular detection of TMV-GFP in mycelia of *V. dahliae* by RT-PCR. Amplicons of the expected size were obtained from total RNA extracted from *V. dahliae* mycelium exposed to viral co-incubation and collected at 6, 9 and 13 dpi. PCR assay of GFP, CP and RdRp of TMV yielded the expected amplified product of 466 bp, 643 bp and 241 bp, respectively. Amplicons were absent in mock-inoculated mycelia of *V. dahliae*, used as negative control; M = HyperLadder^TM^ 100bp (Bioline, Meridian Bioscience Inc., Cincinnati, OH, USA); w = white control (no cDNA); − = negative control from mock-inoculated mycelia; + = *V. dahliae* with TMV-GFP.

RT-PCR consistently confirmed the presence of replicating TMV-GFP in *B. cinerea* (Figure 3) and *V. dahliae* (Figure 4) cells, and its absence in Fol and *M. fructicola*, as well as in mock-inoculated cultures, served as negative controls. Total RNA extracted from mycelia collected at 6, 9, and 13 dpi was used to target the GFP, the CP of TMGMV, and the RdRp of TMV-1056, yielding the expected amplified products of 466 bp, 643 bp, and 241 bp, respectively, in all the samples collected at each sampling time.

Additionally, Northern blot analysis of total RNA extracted from mycelial samples of *V. dahliae* (Figure S1a) and *B. cinerea* (Figure S1b) exposed to viral co-incubation and harvested from liquid cultures at 13 dpi provided further evidence of viral replication through the detection of genomic RNA(gRNA) of TMV-GFP with the 241 bp-long TMV-RdRp DIG-labelled DNA probe (Figure S1a) and the detection of subgenomic RNA (sgRNA) of CP corresponding to the RNA sequence replicated by the TMV CP promoter (Figure S1b). In both cases, gRNA was also detected in *N. benthamiana* TMV-infected plants, used as a positive control, but not in mock-inoculated tobacco plants.

### 3.2. Temporal Dynamics of TMV RNA Accumulation in Mycelia of Verticillium and Botrytis spp.

**Figure 5:**
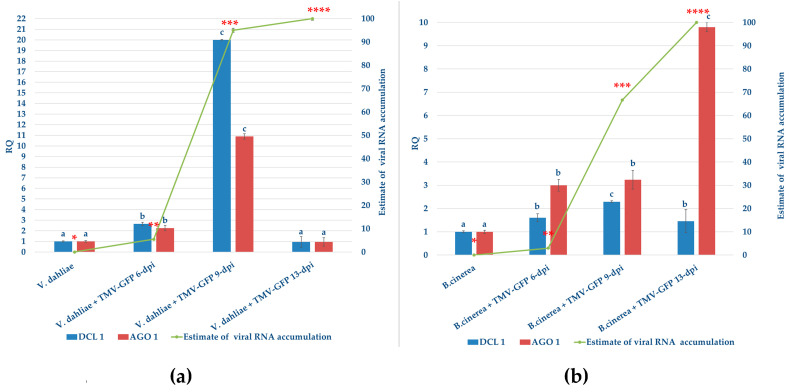
TMV-GFP RNA accumulates in the mycelia of *V. dahliae* and *B. cinerea*, eliciting host RNAi responses. Viral RNA levels (green lines) were quantified by RT-qPCR and are presented as the mean of two independent experiments. Samples were collected from liquid cultures of *V. dahliae* (**a**) and *B. cinerea* (**b**) at 6, 9, and 13 days post-inoculation (dpi) with TMV-GFP. Each point on the line chart represents the average of three biological replicates per experiment. Different numbers of asterisks (*) indicate statistically significant differences according to one-way ANOVA followed by Tukey’s post hoc test (*p* < 0.05). Vertical error bars represent the standard error of the mean (SEM). The figures also include bar charts showing the relative quantity (RQ) of DCL1 (blue bars) and AGO1 (red bars) transcripts in *V. dahliae* (**a**) and *B. cinerea* (**b**) and are presented as the mean of two independent experiments. Total RNA was extracted from mycelia exposed or not exposed to TMV-GFP at 6, 9, and 13 dpi. Gene expression values were normalized to β-tubulin (*V. dahliae*) and actin (*B. cinerea*) as reference genes. Bars represent mean RQ values from three biological replicates per experiment. Different letters (a–c) indicate statistically significant differences (one-way ANOVA with Tukey’s post hoc test, *p* < 0.05). Vertical error bars represent SEM across replicates.

The progression of viral RNA accumulation was monitored by RT-qPCR using total RNA extracted from *V. dahliae* and *B. cinerea* mycelia exposed or not to TMV-GFP infection. The mycelium was collected from liquid cultures at 6, 9, and 13 dpi (Figure 5).

The time points were selected based on previous reports of TMV infection in host plants such as *Nicotiana benthamiana*, where it has been shown that early replication and local infection typically occur within the first 3–6 days post-infection (dpi), while systemic movement and peak viral accumulation often occur between 9 and 14 dpi [43,54].

In our study, since our aim was to evaluate whether TMV could replicate and persist in fungal mycelium, we focused on intermediate time points (6 and 9 dpi) where there is a transition from the initial to the intermediate replication phase, and 13 dpi to evaluate potential persistence or systemic spread within fungal colonies after prolonged exposure. At these same time points, the host RNAi response was also observed over time, correlating changes in the gene expression of DCL1 and AGO1 with the potential viral load.

In *V. dahliae*, TMV RNA levels quickly increased 20-fold between 6 and 9 dpi, followed by a further 1-fold increase between 9 and 13 dpi. This resulted in an overall 22-fold increase during the analyzed time course. Specifically, TMV RNA was detected at the lowest level at 6 dpi with approximately 1.1-fold (Figure 5a). A similar viral accumulation pattern was observed in *B. cinerea* mycelia infected with TMV-GFP. However, compared to *V. dahliae*, the increase in viral load in *B. cinerea* was slighter, showing a 6.6-fold increase between 6 and 9 dpi and a 3.4-fold increase between 9 and 13 dpi (Figure 5b).

Overall, TMV RNA accumulation in *B. cinerea* was 12-fold lower than in *V. dahliae* at the same time points, consistent with the results obtained from Northern blot analysis (Figure S1), potentially due to biological, molecular or structural differences, such as cell wall composition, receptor compatibility [62] between these two fungal species. Furthermore, this difference would also depend on the different survival strategies used, such as asexual spores (from *B. cinerea*) and microsclerotia (from *V. dahliae*) [63,64,65].

Their different ecological roles and infection strategies could suggest, along with our findings, the reason for a distinct yet progressively increasing accumulation of TMV in phylogenetically distant plant-pathogenic fungi, such as *Verticillium* and *Botrytis* spp.

### 3.3. Cross-Kingdom Viral Infection Activates RNA Interference Machinery and siRNA Biogenesis in Verticillium and Botrytis spp.

To investigate the host RNA response of *V. dahliae* and *B. cinerea* to TMV-GFP infection, we used RT-qPCR to quantify the relative abundance of two major RNAi hallmark transcripts: DCL1 and AGO1, which are involved in the recruitment of 21-nt virus-derived small interfering RNAs (vsiRNAs), assembly of the RNA-induced silencing complex (RISC), and dissemination of the silencing signal. The selection of DCL1 and AGO1 was further motivated by the ability of the TMV silencing suppressor protein p122 to inhibit RNAi, primarily through its preferential binding to 21-nt double-stranded vsiRNAs, thereby interfering with RISC assembly [66].

The transcription profile was assessed using total RNA extracted in triplicate from mycelial samples collected at 6, 9, and 13 dpi. The specificity of reactions was confirmed by melting curves.

Compared to mock-inoculated controls, TMV-GFP infection in *V. dahliae* induced the up-regulation of the *DCL1* and *AGO1* transcripts of only 1.5-fold at the first sampling time, which is consistent with a 1.2-fold increase in viral load (Figure 5a). Interestingly, a significant upregulation was observed at 9 dpi, with a 20- and 10-fold increase in the abundance of *DCL1* and *AGO1* gene transcripts, respectively. This displayed a substantial correlation between viral RNA accumulation in *V. dahliae* and the relative RNAi response (Figure 5a). Although the viral load remained high at 13-dpi, the abundance of *DCL1* and *AGO1* gene transcripts decreased to values similar to those recorded in the mock-inoculated *V. dahliae* control. Upregulation of *DCL1* and *AGO1* was also observed in TMV-GFP infected *B. cinerea* samples, even if the transcriptional induction recorded was less marked and more gradual (up to 3-fold increase from 6 to 9 dpi, and up to 6.6-fold increase from 9 to 13 dpi, for AGO1, and 1.6-fold at 6 dpi and 2.2-fold at 9 dpi for DCL1 (Figure 5b). There was a significant correlation with both viral RNA accumulation and the relative abundance of the transcripts. To elucidate the connection between the significant modulation of *DCL1* and *AGO1* genes following TMV infection in *V. dahliae* and *B. cinerea*, we investigated the accumulation of siRNAs that matched the target gene sequences.

Therefore, we attempted to isolate vsiRNAs from total RNA preparations extracted from *V. dahliae* and *B. cinerea* mycelia with TMV-GFP at 13 dpi. Northern blot analysis using a DIG-labelled RNA probe specific for the CP of TMV-GFP detected vsiRNAs unique to TMV in infected mycelia but not in mock-inoculated wild-type counterparts (Figure S2), although the signal was very weak. The vsiRNAs showed the same mobility as the 21-nt single-stranded (ssDNA) primer marker.

### 3.4. TMV Infection Persists in Plated Subcultures

**Figure 6:**
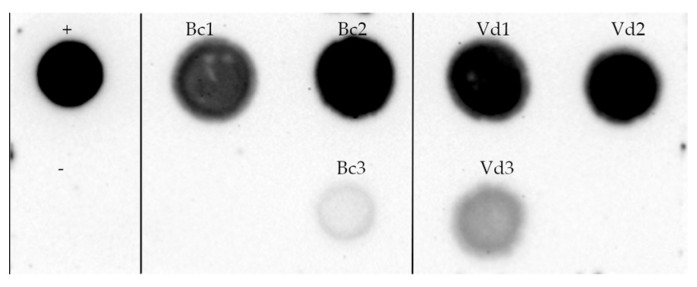
Dot-blot hybridization with a DIG-labelled DNA probe against TMV-GFP RNA of total RNA (5 μg) extracted from sub-cultured mycelia samples of *V. dahliae* (Vd1, Vd2, Vd3) and *B. cinerea* (Bc1, Bc2, Bc3) collected at 13-dpi and grown individually on solid medium (PDA) for 10 days. +, positive control of 50 ng of pTMVGFP1056CP plasmid); −, mycelia sample not exposed to incubation with TMV.

To assess the stability of TMV infection in *V. dahliae* and *B. cinerea* mycelia, monoconidial cultures were collected at 13 dpi from the liquid medium and transferred to a solid PDA substrate for the first subculture. The resulting mycelia were treated with sodium hypochlorite before total RNA extraction, and RNA was blotted onto nylon membrane and hybridized with a DNA probe specific for the CP gene of TMV-GFP. Figure 6 shows a clear hybridization signal in all blots of *V. dahliae* and *B. cinerea* subcultures exposed to viral inoculum.

### 3.5. Pathogenicity, Morphology, and Growth Rate of Verticillium and Botrytis spp. Remain Unaffected by TMV

**Figure 7:**
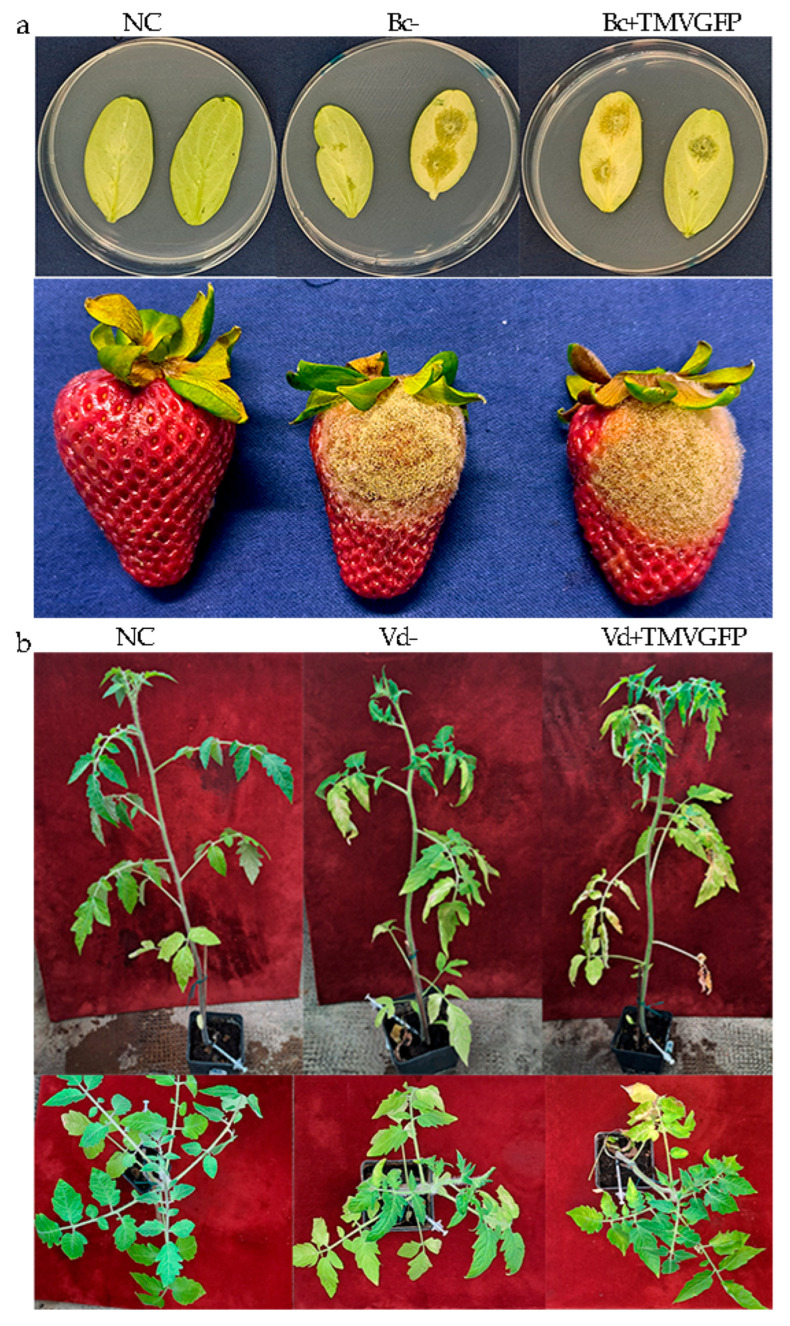
TMV-GFP infection does not affect the pathogenicity of *B. cinerea* and *V. dahliae* on cucumber leaves, strawberry fruits, and tomato plants. (**a**) Pathogenicity test performed on cucumber leaves and strawberry fruits (**a**) showed no significant difference in the diameter of the lesions induced by 10^5^/mL conidial suspension of *B. cinerea* challenged with TMV-GFP (Bc+TMVGFP) or not exposed to TMV inoculum (Bc−. Fruits inoculated with sterile distilled water containing 0.01% Tween 20 and cotyledons inoculated with sterile PDA plugs were used as negative controls (NC). Pathogenicity test on *S. lycopersicum* cv Marmande plants (**b**) inoculated with 10^7^/mL conidial suspension of *V. dahliae* transfected with TMV-GFP (Vd+TMVGFP) or not exposed to TMV inoculum (Vd−) showed no significant differences in symptoms between the two inocula. Disease symptom intensity was characterized by leaf yellowing, necrosis, and wilting. Tomato plants inoculated with PBS buffer served as a negative control (NC).

We investigated the impact of viral infection on the pathogenicity of *V. dahliae* and *B. cinerea* using virulence assays. Cucumber cotyledons, organic strawberry fruits, and tomato cv Marmande plants were inoculated with conidial suspensions of the first subculture of *B. cinerea* and *V. dahliae* at the concentration of 10^5^/mL and 10^7^/mL, respectively. In each experiment, fruits inoculated with sterile distilled water, or cotyledons inoculated with sterile PDA plugs, and tomato plants inoculated with PBS buffer were used as negative controls. The samples were either transfected with TMV-GFP or not. The TMV-GFP infection was verified by dot blot hybridization (Figure 6). Following inoculation, both the wild-type and the TMV-infected *Botrytis* inoculum induced typical symptoms in cucumber cotyledons and organic strawberries, consisting of soft, water-soaked lesions that quickly expanded into necrotic areas. These lesions were often covered with dense grey fungal growth, eventually leading to tissue breakdown and collapse (Figure 7a). The severity and size of the lesions increase over time and until the end of the lesion development (5-dpi) (Table 1). Cucumber leaves and strawberries rubbed with a mixture of sterile PDA plugs and sterile distilled water remained symptom-free. Lesion diameters detected at 5 dpi were very similar for both cultures, suggesting that the presence of TMV in *B. cinerea* did not significantly alter its pathogenicity.

The pathogenicity test on tomato plants inoculated with *V. dahliae* was extended to 36 days to allow for systemic spread within the plants (Figure 7b). Disease symptoms primarily consisted of wilting of the lower leaves, yellowing and lesions on the leaf margins within 5 weeks after inoculation. Also in this case, no significant differences in pathogenicity of *V. dahliae* were detected between virus-free and TMV-GFP-transfected *Verticillium* (Table 2).

**Figure 8:**
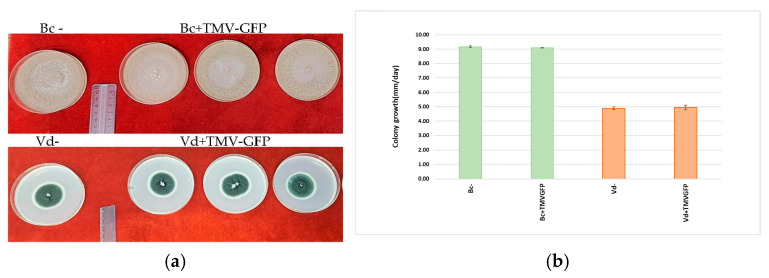
Effect of plant virus infection on morphology and growth rate of *B. cinerea* and *V. dahliae*. Morphology of *B. cinerea* (Bc+TMV-GFP) and *V. dahliae* (Vd+TMV-GFP) mycelia transfected with TMV-GFP, compared with wild-type mycelia (Bc− Vd−) after 10 days of growth on PDA plates, is unaffected by virus transfection (**a**). Colony growth of *B. cinerea* (green bars) and *V. dahliae* (orange bars) challenged or not with TMV-GFP, and expressed as mm/day, shows no statistically different data, according to one-way ANOVA analysis followed by Tukey’s HSD test (*p* ≤ 0.05). Vertical bars represent the SEM calculated on five samples for each condition (**b**).

The morphology and growth rate of *B. cinerea* and *V. dahliae* mycelia, both wild type and those transfected with TMV-GFP, were assessed for 10 days of growth on PDA plates. Neither the morphology of mycelia (Figure 8a) nor the growth rate of cultures (Figure 8b) was altered noticeably by TMV. Data were analyzed using analysis of variance. Means and standard deviation were not significantly different (*p* ≤ 0.05), according to the Tukey HSD post hoc test.

## 4. Discussion

This work improves our understanding of the recently discovered ability of plant viruses, such as TMV, to infect phytopathogenic fungi belonging to different *taxa*, such as *Botryotinia* and *Verticillium* [51,52]. These fungi are characterized by different modes of transmission, hyphal morphology and size, genomic organization, and plant pathogenesis [67,68]. This suggests that plant viruses can effectively cross traditional species barriers and successfully infect certain fungal organisms. Natural cross-kingdom infections have been increasingly documented. For example, cucumber mosaic virus (CMV) has been shown to naturally infect the basidiomycete *Rhizoctonia solani*, with bidirectional transmission occurring between the plant and fungi. Notably, CMV infection enhanced the virulence of *R. solani* following transfection [69]. Scientific evidence suggests that certain fungal species belonging to different orders, such as *Hypocreales* and *Pleosporales*, can naturally acquire plant viruses from their hosts during the colonization process. In this way, they act as “temporary reservoirs” for viruses like CMV, Turnip mosaic virus (TuMV), and Broad bean wilt virus 2 (BBWV2), and then transmit them non-persistently to other host species [13]. We conducted a detailed investigation of TMV interactions with several taxonomically different plant fungi using an integrated approach combining microscopy observations, molecular assays, and phenotypic analyses. Our findings provide evidence that TMV can enter, replicate, and persist in *B. cinerea* and *V. dahliae*, establishing them as its host and transmitting the viral infection from one generation to another through their conidia, at least for the first subculture.

On the contrary, we observed that TMV transfection failed in Fol and *M. fructicola*, suggesting that, unlike in plants, in filamentous fungi, there may be a more limited or different basis for host–virus specificity of plant virus infection.

The fungal cell wall is a physical barrier that can impede virus entry. It is composed mainly of chitin, β-glucans, and glycoproteins, and its architecture and thickness vary between species. As specifically reported for mycoviruses, this rigid structure is generally impermeable to large macromolecules [70,71]. Differences in cell wall composition or porosity among fungi might explain the host range: if *Fol* and *M. fructicola* have less-permeable cell walls, TMV particles may not efficiently penetrate or be taken up, unlike in *B. cinerea* and *V. dahliae*. Indeed, the lack of an extracellular phase for most mycoviruses is attributed to the “impenetrable” fungal cell wall, which blocks virus uptake [70]. We hypothesize that *Fol* and *M. fructicola* likely present a tough barrier to TMV entry.

Confocal and epifluorescence microscopy observations demonstrated that TMV-GFP exclusively infects and replicates in *V. dahliae* and *B. cinerea* via transcription and expression of sgRNAs of the GFP (Figure 2), with no ectopic expression of TMV-GFP detected in *F. oxysporum* and *M. fructicola* when challenged with TMV. The ability of *V. dahliae* and *B. cinerea* to become infected by TMV was confirmed by RT-PCR, which targeted three viral genomic regions: GFP, CP and viral RdRp (Figure 3 and Figure 4). This was also confirmed by Northern blot analyses, which were specific for the detection of the viral CP of TMGMV (for *B. cinerea*) and TMV RdRp (for *V. dahliae*) (Figure S1). TMV genomic RNA was exclusively present in *V. dahliae* and *B. cinerea*, whose mycelia were preliminarily subjected to sodium hypochlorite treatment before RNA extraction and analysis, to effectively degrade viral particles and encapsidated RNA [25,27]. These findings suggest that TMV can establish productive infections in certain filamentous fungi, and that replication may depend on host-specific biological factors such as cell wall permeability, the presence of compatible surface receptors, or the availability of intracellular conditions favourable for viral replication [72,73].

Unlike plant cells, which viruses enter via wounds or vectors, fungal cells do not have known specific receptors for plant viruses. We speculate that TMV’s successful entry into *B. cinerea* and *V. dahliae* could rely on non-specific uptake mechanisms (such as endocytosis or fungal cell wall pores) that might not be effective in *Fol* or *M. fructicola*. Moreover, host-specific factors—such as the presence (or absence) of compatible surface molecules or cellular entry pathways—could determine whether TMV can internalize and initiate infection. It is well known that the ability of a plant virus to infect a fungus can depend on the fungus’s cellular environment. For example, certain fungi only become susceptible to plant viruses when specific conditions are met (e.g., cell wall is made more permeable or typical defences are suppressed). Thus, a mismatch in virus–host cell interactions (entry and uncoating processes) may render Fol and *M. fructicola* non-permissive for TMV.

The fungal RNA interference as a defence mechanism is a key factor in modulating the replication of the viruses inside the host cell. RNA silencing is a conserved defence mechanism in fungi that can recognize and degrade foreign viral RNA, as clearly demonstrated for several mycoviruses [74,75]. Therefore, it could be hypothesized that Fol and *M. fructicola* possess a very effective silencing response that aborted TMV replication early. Although in our experiments we could not detect TMV gene expression in these two fungi, the importance of RNAi was evident from parallel cases in the literature [75]. Therefore, a robust RNAi response likely serves as a critical barrier to TMV in *Fol* and *M. fructicola*.

These considerations are supported by previous findings where *Fusarium graminearum* supports only limited TMV replication unless its RNA silencing pathway is suppressed [76]. Co-infection with the mycovirus cryphonectria hypovirus 1 (CHV1) led to higher TMV accumulation, due to the CHV1’s suppressor proteins attenuated the host’s gene silencing machinery [14,77,78,79].

These findings emphasize that Fol (a related *Fusarium* species) and *M. fructicola* likely resist TMV through strong RNAi activity. And suggest that the fungal RNAi pathway acts as a critical barrier to viral replication. Consistently, when TMV infects a permissive fungus, the fungus’s Dicer and Argonaute genes are induced, as observed in our *B. cinerea*/*V. dahliae* results and reported previously in *P. infestans* [26]. Indeed, it has been demonstrated that TMV infection in the oomycete triggers an RNAi-based response by upregulating key genes involved in this pathway [26]. The absence of TMV replication in Fol and *M. fructicola* suggests that the virus was either rapidly degraded by such antiviral RNAi or otherwise unable to overcome the host’s silencing defences. Based on these mechanisms (cell wall architecture, entry factor compatibility, and RNA silencing), it could be possible to hypothesize that Fol and *M. fructicola* likely restrict TMV. If TMV can infect and persist in certain soil- or plant-associated fungi, those fungi might serve as alternative hosts or “reservoirs” for the virus in nature [7,13,79]. Nevertheless, fungi could harbour plant viruses in the absence of plant hosts, allowing the virus to overwinter or survive between crop seasons in soil or decaying plant matter. This is supported by recent findings: for example, Cao et al. [13] showed that multiple plant viruses can transiently infect diverse fungi, suggesting that fungi (alongside plants and insect vectors) could be living sources of plant viruses in nature [7,14]. The ability of TMV to enter a fungus expands its host range and indicates the virus might hide in fungal communities without causing obvious symptoms. Such fungal reservoirs could complicate disease management—a virus might linger cryptically in a non-plant host and later reinfect crops. This possibility raises new questions about virus epidemiology, as controlling a plant virus may require considering fungal hosts in the environment. However, data by Cao et al. [13] demonstrates that after prolonged maintenance of the fungal culture in the laboratory, many of the fungal strains have lost the virus. These observations suggest that the nonpersistent acquisition of plant viruses by fungi may commonly occur in nature.

While plant viruses traditionally spread via insects, nematodes, or mechanical means, recent studies propose that fungi can act as biological vectors for plant viruses [13,80]. When a fungus colonizes a plant (e.g., *V. dahliae* infecting roots or *B. cinerea* infecting leaves), any virus harboured in the fungal mycelium might be delivered into plant cells. There is evidence supporting this bidirectional exchange: for instance, research has documented viruses moving from plants to fungi and vice versa under natural conditions [13]. Overall, these findings illustrate how fungi–virus associations could serve as a bridge between plant and fungal disease cycles. Although TMV transmission via fungal infection has not been definitively shown in agriculture, it cannot be completely excluded a theoretical scenario where a TMV-infected *V. dahliae* might introduce the virus into a tomato’s vascular system during wilt infection, or *B. cinerea* could deposit virus particles on the surface of an infected fruit. Although this hypothesis remains purely speculative, it underscores a new dimension in plant virus epidemiology.

Based on these observations, our study aimed to investigate the intracellular dynamics of TMV within *B. cinerea* and *V. dahliae* by quantifying the temporal progression of viral RNA accumulation and assessing the corresponding host RNAi-mediated defence response to infection. Temporal tracking of viral RNA accumulation in *V. dahliae* and *B. cinerea* by RT-qPCR revealed that TMV was replicating rapidly in both fungi, reaching high titers at 13-dpi (Figure 5). In *V. dahliae* we observed a biphasic trend in viral RNA accumulation based on a slight increase at 6 dpi, followed by a pronounced peak up to 20-fold increase at 9 and 13 dpi (Figure 5a). Conversely, *B. cinerea* appears to defend itself more strongly and effectively than *V. dahliae*, limiting virus replication at some levels, which still increased up to 10-fold change at 9 and 13 dpi, albeit more gradually and moderately (Figure 5b). This suggests that *V. dahliae* cells may offer a more permissive environment or exhibit a less robust RNAi response. Supporting this observation, a gene expression study of DCL1 and AGO1 transcripts, two key RNA interference (RNAi) genes, was performed in *V. dahliae* and *B. cinerea* during all time courses analyzed, revealing a significant activation of antiviral silencing pathways in both fungi while harbouring TMV. Prolonged viral exposure may lead to a reduction in transcriptional factors or strong downregulation of RNAi-related genes due to intense RNAi activity. Although multiple DCL and AGO genes are present in many filamentous fungi, the specific contributions of each paralog to antiviral RNAi remain largely unresolved for most species and were not examined in this study. Previous studies in *F. graminearum* and *C. parasitica* suggest a predominant role for DCL2 and AGO1/2 homologs [74,79]; however, functional characterization in *B. cinerea* and *V. dahliae* remains to be conducted.

Whenever TMV (or a similar plant virus) replicates in a fungus or oomycete, the host typically activates an RNA silencing response. In our study, both *B. cinerea* and V*. dahliae* upregulated DCL1and AGO1 genes in response to TMV infection, producing virus-derived siRNAs—clear evidence of the RNAi defence at work. These results are completely in accordance with previous findings on *P. infestans* which upregulated RNA-silencing genes when infected by TMV [26]. The fact that RNAi response is common in distantly related organisms (*B. cinerea* and *V. dahliae* and *P. infestans* oomycete) is a similarity indicating an evolutionarily conserved defence mechanism. RNAi-based antiviral defence is observed across multiple fungal species: in *C. higginsianum*, deletion of DCL1 and AGO1 derepressed resident dsRNA viruses and caused developmental defects [81]. Similarly, in *F. graminearum*, TMV infection triggers the production of virus-derived siRNAs, and mutants lacking DCL1 or DCL2 accumulate far fewer vsiRNAs, highlighting the central role of the RNAi machinery in degrading foreign viral RNA [82].

In our study, TMV–*V. dahliae* interactions appeared to be in dynamic equilibrium. This was characterized by alternating phases of DCL1 and AGO1 host-mediated RNAi activation in the host at 6 and 9 dpi, followed by down-regulation at 13 dpi, likely due to the suppressive activity of the TMV 122 kDa RdRp subunit (p122), which acts as a viral silencing suppressor protein (SRS) [66,83,84,85,86]. This modulatory mechanism may promote TMV persistence at high titers in *V. dahliae* up to 13 dpi, despite the significant decrease in DCL1 and AGO1 transcripts observed from 9 to 13 dpi. Prolonged TMV infection and sustained RNAi activity could lead to transcriptional exhaustion or active RNAi suppression by p122-SRS, which binds vsiRNAs and interferes with RISC assembly [85]. The role of viral suppressors in modulating RNA silencing within fungal cells is well established. Many mycoviruses encode suppressors of RNA silencing (SRS) that promote viral replication and persistence of infection [76]. For instance, the ORF2 protein of *F. graminearum* virus 1 acts as a suppressor of the DCL2 and AGO2 enzymes’ transcriptions, compromising the host’s antiviral defence mechanisms [86]. Similarly, *Botrytis* virus F (BVF) suppresses the expression of key genes involved in the RNAi machinery, such as DCL1 and DCL2, during the early stages of infection in *B. cinerea* [87]. Taken together, these findings support the hypothesis that the viral suppressor p122 acts as a dynamic modulator of fungal RNAi activity, facilitating viral stability and replication within the host organism.

Conversely, slight modulation of DCL1 and AGO1 transcripts was observed in *B. cinerea* during TMV infection when moving from 6 to 9 dpi, concomitant with a significant 6-fold increase in viral titer. However, the marked upregulation of AGO1-RNAi-mediated defence transcript was observed from 9 to 13 dpi, which was congruent with the decrease in viral titer increase rate, as AGO1 is a well-known secondary component of the RNAi pathway. *V. dahliae* and *B. cinerea* response to TMV infection was further confirmed by the detection of vsiRNAs (Figure S2), highlighting the evolutionary conservation of antiviral RNA silencing mechanisms across eukaryotic taxa [74]. Thus, the results of our study are consistent with the observed upregulation of DCL1 and AGO1 genes in *V. dahliae* and *B. cinerea*, albeit to different degrees of intensity, which is likely due to their different taxonomic diversity. This upregulation may result from the inability of the TMV p122 suppressor of RNA silencing (SRS) to effectively inhibit RNA interference (RNAi), leading to the increased production of virus-derived small interfering RNAs (vsiRNAs). These findings emphasize the intricate co-evolutionary arms race between viral pathogens and fungal hosts, wherein viral suppressors such as p122 play a pivotal role in overcoming host defences [74]. Understanding these dynamics is essential not only for elucidating virus–fungus interactions but also for exploring RNAi-based disease control strategies. However, if RNAi components become downregulated or exhausted over time, the efficacy of RNAi-mediated antiviral interventions may be compromised. In this context, it is interesting to note that *V. dahliae* appears to use RNAi not only as a defence mechanism against TMV, but also to regulate its virulence. Jin et al. [46] identified the ability of *V. dahliae* to produce a micro-RNA (milRNA), VdmilR1, which silences the VdHy1 gene, which plays a pivotal role in regulating the fungus’s infectivity towards host plants. This evidence highlights that the RNAi pathway extends beyond antiviral defence, serving as a sophisticated regulatory tool that fine-tunes fungal aggressiveness by balancing growth and virulence, thereby profoundly impacting the dynamic infection and host resistance. Similarly, the closely related species, *Verticillium nonalfalfae*, has been deeply characterized as a fully functional RNAi system, with key genes DCL, AGO, and RDRP markedly upregulated during plant infection [40]. These observations suggest that, within the *Verticillium* genus, RNAi fulfils dual roles: safeguarding genomic integrity by defending against viral pathogens and regulating virulence-associated genes through both epigenetic and PTGS mechanisms. Future research focusing on proteomic alterations and the long-term modulation of RNAi pathways in fungal pathogens will be crucial to unravelling the full complexity of these regulatory networks. The presence of a plant virus in a fungal pathogen could alter the course of plant disease. In our study, TMV infection did not affect the growth or colony morphology of *V. dahliae* and *B. cinerea* (Figure 8) nor did it impact their in vivo pathogenicity on cucumber leaves, strawberry fruit or tomato plants (Figure 7; Table 1 and Table 2). These fungi were just as pathogenic with or without the virus. The virus remained stable even after the first subcultures, suggesting vertical transmission and persistence within fungal populations without compromising host fitness. This neutrality is noteworthy, and it can be related to similar reports (e.g., TMV-infected *C. acutatum* showed no change in growth, morphology, or pathogenicity) [27]. In that study, the virus could persist in the fungal mycelium without detectable detriment to the fungus, and *C. acutatum* did not exhibit any growth defects or loss of virulence when infected by TMV. This indicates a common pattern: certain filamentous fungi can support a productive TMV infection while essentially tolerating the virus. This similarity indicates that TMV’s host range can extend to multiple fungi, at least within the Ascomycetes, under the right conditions. However, cases where cross-kingdom virus infection affects disease severity have also been reported.

Mascia et al. [26] reported that TMV was able to replicate in *P. infestans* and produced a phenotypic effect: the virus-infected oomycete grew more vigorously (producing more mycelium and sporangia) yet caused milder disease on plants compared to virus-free *P. infestans*. Unlike *P. infestans*, our *V. dahlia*e and *B. cinerea* showed no change in virulence due to TMV, and no growth promotion either. This side-by-side comparison underlines that cross-kingdom viral infections can have variable impacts on the host organism. Differences in the host’s biology (fungus vs. oomycete) or in how the host’s immune response interacts with TMV might explain why *P. infestans* responded differently.

To further broaden the comparison, the case of *R.solani* can also be considered. Andika et al. [7] found that *R. solani* infected with CMV became more aggressive. In contrast, our TMV-infected fungi did not change aggressiveness, underscoring that different virus–fungus combinations yield different interactions. Additionally, while *F. graminearum* is not one of the fungi we tested, it provides a useful comparison for host compatibility. TMV can enter *F. graminearum* but replicates poorly on its own, likely due to strong antiviral defences, as reported by Bian et al. [14]. Only when those defences are suppressed (e.g., by co-infecting with a mycovirus), does TMV replicate robustly in this fungus. This suggests that *Fusarium* species, in general, might be less permissive hosts for TMV compared to the other fungi (perhaps due to a more potent RNAi response or other factors). Meanwhile, *C. acutatum*, *V. dahliae*, *B. cinerea* and *P. infestans* appear to be more permissive hosts where TMV can at least establish an infection. These examples illustrate that a plant virus can modulate a fungal pathogen’s behaviour in diverse ways, sometimes attenuating disease, sometimes exacerbating it, or having no effect at all. This variability means TMV-infected fungi could subtly influence plant disease dynamics (for instance, by altering the timing or severity of symptoms), even if our specific isolates showed no change. In summary, the ability of TMV to infect fungi has potential ecological and agricultural ramifications: fungi may act as virus reservoirs or novel vectors, and such cross-kingdom interactions could influence the epidemiology of both the viral disease and the fungal disease in crops [13]. Our investigation covered only four fungal species, all of which are filamentous Ascomycetes. This is a small sample of the vast fungal kingdom, as Basidiomycete fungi, obligate biotrophs, or other Oomycetes were not examined. The TMV replication in two of the four species tested may not be generalizable to all fungi. The host range of TMV across fungi could be broader or narrower than our four test species suggest. In particular, because our selection focused on plant-pathogenic fungi, primarily those affecting crops, the outcomes might differ in endophytic fungi or fungi from other environments. Further studies on a wider range of fungi (including additional taxa and lifestyles) are needed to fully understand how common or rare cross-kingdom infections are.

TMV remained detectable after the first subculture, which suggests the virus can be vertically transmitted to new hyphae/conidia in the short term. However, we did not investigate whether the virus continues to be maintained in the fungal populations over multiple successive subcultures. The long-term stability of TMV in these fungi is unknown. Recent research found that although plant viruses can sometimes transmit through fungal spores, they may eventually be lost after repeated subculturing in laboratory conditions [14,70]. Moreover, Cao et al. [13] observed that certain plant viruses infected fungal strains transiently but were not stable after more than six rounds of subculture, possibly because the lab conditions lacked factors needed for persistent infection. Our experiments spanned a limited timeframe, and it is possible that TMV might gradually be cured from *B. cinerea* or *V. dahliae* over many generations, or conversely, that it might integrate stably. Therefore, future work should examine the heritability of such cross-kingdom infections (e.g., whether TMV is still present after numerous subcultures or after fungal sporulation). In conclusion, the results of this study reveal a complex interplay between TMV and in *B. cinerea*, *V. dahliae*, Fol and *M. fructicola*, involving host specificity, RNAi defence mechanisms, and stable viral maintenance without detrimental effects. From an ecological and evolutionary perspective, the ability of plant viruses to infect fungi expands our understanding of viral host ranges and could indirectly affect plant disease dynamics. Therefore, our research contributes to the growing body of knowledge on these complex virus–fungus relationships, which remain largely unexplored [13].

## Figures and Tables

**Table 1:** Pathogenicity test on cucumber cotyledons and strawberry fruits inoculated with a mycelium plug (4 mm diameter) or a suspension of 10^5^/mL conidia of *B. cinerea*, wild type or exposed to TMV-GFP inoculum.

Sample	Lesion Size on Cucumber Leaves, cm	Lesion Size on Strawberry Fruits, cm	N° Total Cucumber Leaves	N° Total Strawberry Fruits
Bc−	2.11 ± 0.28 ^a,^*	2.61 ± 0.11 ^a,^*	15	30
Bc+TMVGFP	2.43 ± 0.27 ^a,^*	2.49 ± 0.13 ^a,^*	15	30
Negative Control	0.00 ± 0.00 *	0.00 ± 0.00 *	5	10

* Mean ± standard error; values within the same column followed by the same letter are not statistically different at the probability levels *p* ≤ 0.05 according to the Mann–Whitney U test.

**Table 2:** Pathogenicity test on *S. lycopersicum* cv Marmande plants inoculated with a suspension of 10^7^/mL conidia of *V. dahliae* wild type or infected with TMV-GFP. Disease severity was rated using foliar symptoms on a scale of 0–5 in which 0 = no symptoms, 1 = >0 to 10% of the plant showing chlorosis or necrosis, 2 = >10 to 25%, 3 = >25–50%, 4 = >50–75%, 5 = >dead plant. Midpoints for disease severity ranges were calculated.

Sample at 36 dpi	Disease Severity (Scale 0–5)	Disease Severity Index (DSI)	N° Total Plants
Vd−	2.67 ± 0.14 ^a,^*	64.28%	21
Vd+TMVGFP	2.76 ± 0.11 ^a,^*	69.05%	21
Negative control	0.0 ± 0.0 *	0.00%	7

* Mean ± standard error; values within the same column followed by the same letter are not statistically different at the probability levels *p ≤* 0.05 according to one-way ANOVA, followed by Tukey’s HSD test.

## Data Availability

The original contributions presented in this study are included in the article. Further inquiries can be directed to the corresponding author.

## Supplementary Materials

The following supporting information can be downloaded at: https://www.mdpi.com/article/10.3390/jof11090619/s1, Figure S1: Detection of viral RNA by Northern blot hybridization with a DIG-labelled DNA probe in 10 μg of total RNA preparations extracted from mycelia collected from liquid cultures of V. *dahliae* (Vd+) and *B. cinerea* (Bc+) at 13 dpi with TMV-GFP; Figure S2: Detection of virus-specific CP-TMGMVsiRNAs in total RNA preparations from mycelia of *B. cinerea* (Bc+) and *V. dahliae* (Vd+) collected at 13-dpi. Mock-inoculated counterparts (Bc− Vd−) were used as negative controls. Panel Gel red-stained 15% polyacrylamide gel (**a**) and after hybridization with TMV-CP DIG-labelled probe and chemiluminescent detection (**b**). Arrowheads indicate the migration positions of 21-nt ssDNA primers (P) used as size markers (**a**) and of siRNAs (**b**). Table S1: Primer pairs used for RT-PCR, qRT-PCR detection and synthesis of probes in this study.
