# Supplementary material for: Tracking of Tobacco Mosaic Virus in Taxonomically Different Plant Fungi
Source: J Fungi (Basel). 2025 Aug 25;11(9):619. doi: 10.3390/jof11090619 (PMC12470614; doi:10.3390/jof11090619)
Supplement: Supplementary file 1 [file jof-11-00619-s001.zip › Figures S1 and S2.pdf]

Supplementary Material

Figure S1

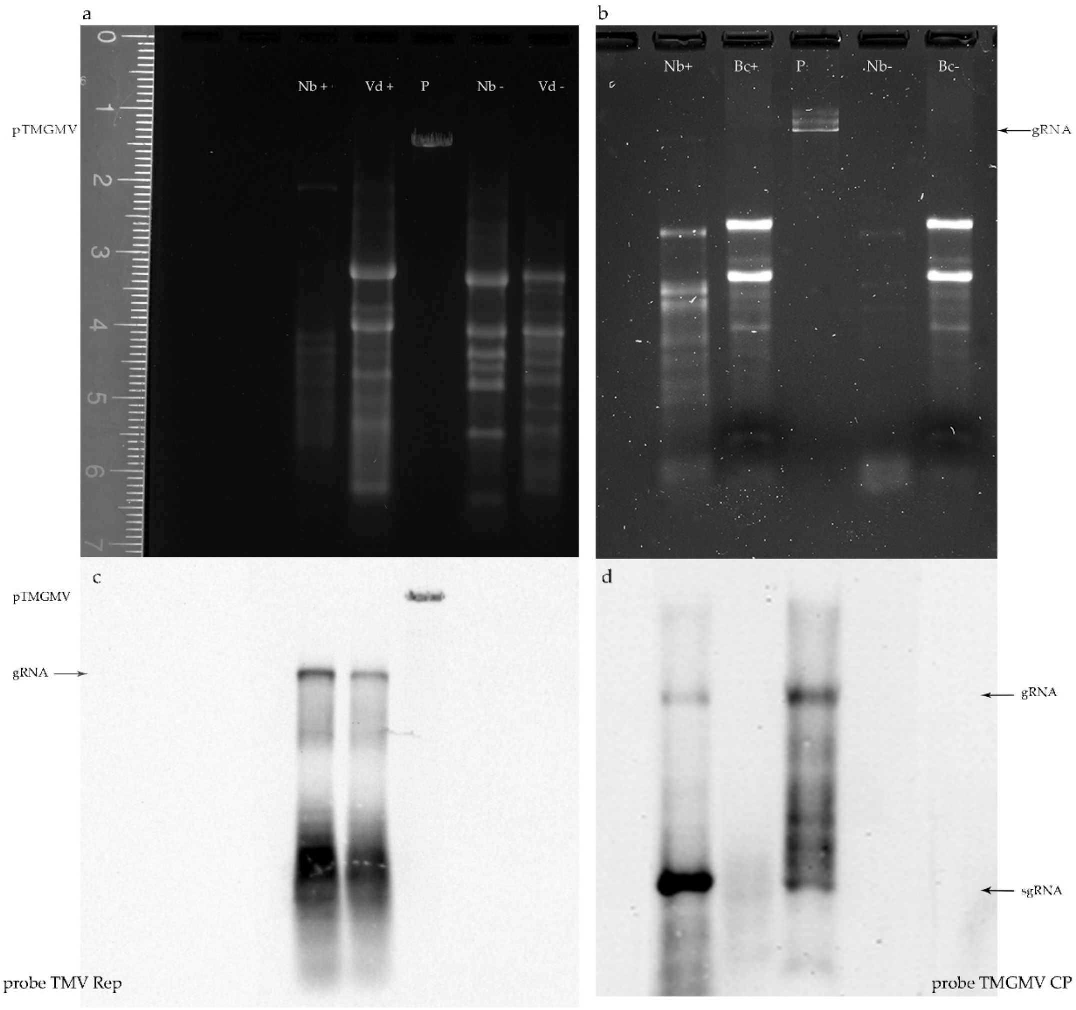

Figure S1. Detection of viral RNA by northern blot hybridization with a DIG-labelled DNA probe in 10 μg of total RNA preparations extracted from mycelia collected from liquid cultures of V. *dahliae* (Vd+) and *B. cinerea* (Bc+) at 13 dpi with TMV-GFP. Total RNA (2 μg) extracted from *N. benthamiana* TMV-infected plants (Nb+) and mock-inoculated plants (Nb-) were used as control. Panel **a** and **b** Gel red- stained 1.2% agarose gel and **c** and **d** after hybridization with TMV-DIG-labelled DNA probe and chemiluminescent detection. (**c**) Abundance of TMV-GFP RNA with DIG-labelled DNA probe for the TMV Replicase. Arrows indicate the TMV genomic (gRNA) RNA. P,=100 ng KpnI-linearized pBSG1057; Vd-,= V. *dahliae* not exposed to co-incubation with viral inocula. (**d**) Abundance of TMV-GFP RNA by northern blot hybridization with a 643-bp DIG-labelled DNA probe for TMGMV CP. Arrows indicate the positions of TMV genomic (gRNA) and coat protein subgenomic (sgRNA) RNAs. P, 200 ng RNA extracted from a purified preparation of TMV-GFP, used as control: Bc-, mycelia of *B. cinerea* not exposed to incubation with TMV.

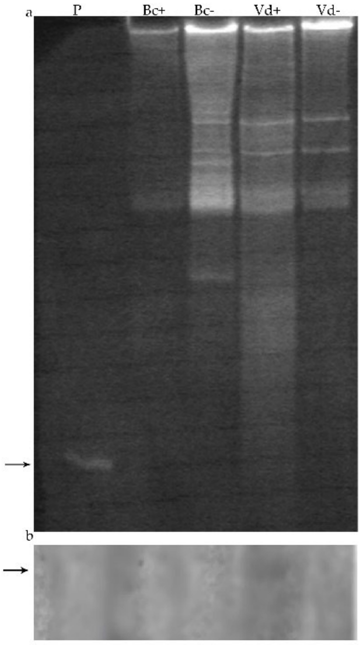

Figure S2. *B. cinerea* and *V. dahliae* mediate the production of vsiRNAs upon viral infection. Detection of virus-specific CP-TMGMVsiRNAs in total RNA preparations from mycelia of *B. cinerea* (Bc+) and *V. dahliae* (Vd+) collected at 13-dpi. Mock-inoculated counterparts (Bc-, Vd-) were used as negative controls. Panel Gel red-stained 15% polyacrylamide gel (**a**) and after hybridization with TMV-CP DIG-labelled probe and chemiluminescent detection (**b**). Arrowheads indicate the migration positions of 21-nt ssDNA primers (P) used as size markers (**a**) and of siRNAs (**b**).
