# Supplementary material for: Tracking of Tobacco Mosaic Virus in Taxonomically Different Plant Fungi
Source: J Fungi (Basel). 2025 Aug 25;11(9):619. doi: 10.3390/jof11090619 (PMC12470614; doi:10.3390/jof11090619)
Supplement: Supplementary file 1 [file jof-11-00619-s001.zip › Table S1.pdf]

Table S1: Primer pairs used for RT-PCR, qRT-PCR detection and synthesis of probes in this study.

Primer	Sequence (5'-3')	Description	Size Product	Refereces
Bc-actin F	TGCTCCAGAAGCTTTGTTCCAA	qRT-PCR Gene Expression	165 bp	Wang, M.; Weiberg, A.; Lin, F.M.; Thomma, B.P.; Huang, H.D.; Jin, H. Bidirectional cross-kingdom RNAi and fungal uptake of external RNAs confer plant protection. Nat. Plants 2016, 2, 16151 . https://doi.org/10.1038/nplants.2016.151.
Bc-actin R	TCGGAGATACCTGGGTACATAG			
Bc-DCL1 F	ACAATCCTATCTTTCGGAAGC	qRT-PCR Gene Expression	110 bp	
Bc-DCL1 R	AGACTCTTCTTCTTGAAGACAG			
Bc-AGO1 F	CATTGGCCCGAGGTGAATTC	qRT-PCR Gene Expression	114 bp	Primers were designed using the Primer3 web interface (Primer3Web version 4.1.0; http://primer3.ut.ee/).
Bc-AGO1 R	TGATGGTGGTTGTTTGCCTG			
VdDCL1-qRT-F	GATGGTACGCGAGAGTGAGA	qRT-PCR Gene Expression	116 bp	Jin, Y.; Zhao, J.H.; Zhao, P.; Zhang, T.; Wang, S.; Guo, H.S. A Fungal milRNA Mediates Epigenetic Repression of a Virulence Gene in Verticillium dahliae. Phil. Trans. R. Soc. B 2019, 374, 20180309. https://doi.org/10.1098/rstb.2018.0309.
VdDCL1-qRT-R	CTGATCCAGTGGGTAACACG			
VdAGO1-qRT-F	ACAAGGATGGCAAGGAAATC	qRT-PCR Gene Expression	136 bp	
VdAGO1-qRT-R	GGTGCAATGGTACAAGCATC			
VdTubulin-qRT-F	ACCTTCGTCGGTAACTCCAC	qRT-PCR Gene Expression	145 bp	
VdTubulin-qRT-R	TGGACTCAGCCTCAGTGAAC			

GFP_Forw	ACTACCTGTTCCATGGCCAACACT	RT-PCR viral detection	466 bp	Mascia, T.; Nigro, F.; Abdallah, A.; Ferrara, M.; De Stradis, A.; Faedda, R.; Minafra, A.; Gallitelli, D. Gene Silencing and Gene Expression in Phytopathogenic Fungi Using a Plant Virus Vector. Proc. Natl. Acad. Sci. USA 2014, 111, 4291–4296. https://doi.org/10.1073/pnas.1400711111.
GFP_ Rev	AAGGGCAGATTGTGTCGACAGGTA			
Coat Protein_Forw	TAAGTAGCCGGAGTTGTGG	Synthesis of CP- probe, RT-PCR viral detection	643 bp	
Coat protein_ Rev	TGAAACTCGAAAGGTTCC			
RdRp_Forw	CCAGCCGATGTCACACATTA	RT-PCR viral detection, TMV RNA accumulation and synthesis of RdRp probe	241 bp	Primers were designed using the Primer3 web interface (Primer3Web version 4.1.0; http://primer3.ut.ee/).
RdRp_ Rev	CATCAGAGTATGTCTCGCCTTG			
